# Carvacrol Modulates Tetracycline Susceptibility and Resistance Gene Expression in Multidrug-Resistant *Salmonella* Typhimurium Strains Isolated from Swine

**DOI:** 10.3390/ani16152362

**Published:** 2026-08-02

**Authors:** Carlos Montero-Palma, Antonio López-Martínez, Antonio Romero-Salmoral, Belén Huerta-Lorenzo, Inmaculada Luque-Moreno, Carmen Tarradas-Iglesias, Alfonso Olaya-Abril, Lidia Gómez-Gascón

**Affiliations:** 1 Animal Health Department, Veterinary Faculty, University of Cordoba, 14014 Cordoba, Spain; v82mopac@uco.es (C.M.-P.); v62rosaa@uco.es (A.R.-S.); sa2hulob@uco.es (B.H.-L.); sa1lumoi@uco.es (I.L.-M.); sa1taigc@uco.es (C.T.-I.); v32gogal@uco.es (L.G.-G.); 2 Zoonotic and Emerging Diseases (ENZOEM), University of Cordoba, 14014 Cordoba, Spain; 3 Biochemistry and Molecular Biology, Science Faculty, University of Cordoba, 14014 Cordoba, Spain

**Keywords:** *Salmonella* Typhimurium, carvacrol, tetracycline, antimicrobial resistance, checkerboard assay, *tetA*, *tetB*, swine

## Abstract

*Salmonella* Typhimurium is an important pathogen affecting both animal production and public health, with pigs representing a major reservoir. The increasing prevalence of antimicrobial resistance highlights the need for alternative or complementary therapeutic strategies. Carvacrol, a phenolic constituent of *Origanum vulgare* essential oil, has recognized antimicrobial activity and may contribute to the modulation of antimicrobial resistance mechanisms. The present study investigated the antimicrobial activity of carvacrol and tetracycline, individually and in combination, against swine-derived multidrug-resistant *Salmonella* Typhimurium strains. Minimum inhibitory concentrations (MICs) and minimum bactericidal concentrations (MBCs) were determined by broth microdilution. Interactions between both compounds were assessed by checkerboard assays using the fractional inhibitory concentration index (FICI). In addition, the modulatory effect of carvacrol on the expression of the tetracycline resistance genes *tetA* and *tetB* was evaluated to provide preliminary insight into the molecular basis of these interactions. Relative expression of tetracycline resistance genes was analysed by quantitative real-time PCR in selected isolates exposed to tetracycline alone or combined with carvacrol. Carvacrol MIC values ranged from 0.5 to 16 mg/mL, with MIC_50_ and MIC_90_ values of 2 and 4 mg/mL, respectively. MBC and MIC values were identical in 84.9% of the isolates, indicating a strong bactericidal effect. Tetracycline MIC_50_ and MIC_90_ values were 256 and 512 µg/mL, respectively. Checkerboard assays showed synergistic interactions in 14.3% of strains, additive effects in 50.0%, indifferent responses in 28.6%, and antagonism in 7.1%. Combined exposure reduced tetA and tetB expression in most tested isolates compared with tetracycline alone, although responses were strain-dependent. These findings demonstrate that carvacrol exhibits antimicrobial activity against swine-derived multidrug-resistant *Salmonella* Typhimurium and may enhance tetracycline activity in a subset of isolates under the experimental conditions evaluated, while modulating resistance-associated gene expression. These findings provide a basis for future transcriptomic and proteomic studies aimed at further elucidating the molecular mechanisms underlying the interaction between carvacrol and tetracycline.

## Simple Summary

*Salmonella* Typhimurium is an important pathogen in pig production and a relevant cause of foodborne disease in humans. The increasing occurrence of antimicrobial resistance, particularly multidrug-resistant strains, has encouraged the search for alternative or complementary control strategies. Carvacrol, a natural compound present in oregano (*Origanum vulgare*) essential oil, has recognized antimicrobial properties. In this study, we evaluated its antimicrobial activity alone and in combination with tetracycline against swine-derived *Salmonella* Typhimurium isolates and explored its effect on tetracycline resistance-associated genes. Carvacrol inhibited bacterial growth in most isolates and, when combined with tetracycline, enhanced antibiotic activity in some strains while reducing the expression of tetracycline resistance genes in selected isolates. Although these findings were obtained under in vitro conditions, they provide a foundation for future mechanistic and in vivo studies to determine whether carvacrol could serve as a natural antimicrobial adjuvant, supporting more prudent antimicrobial use and antimicrobial resistance mitigation in swine production within a One Health framework.

## 1. Introduction

*Salmonella* spp., particularly *Salmonella* Typhimurium, is one of the main gastrointestinal pathogens affecting the swine production sector. Moreover, it represents a major zoonotic and foodborne public health concern, as pigs constitute an important reservoir and may contribute to transmission through contaminated animal-derived products [1,2]. The spread of antimicrobial resistance (AMR) represents one of the major threats to global public and animal health. According to data from the European Antimicrobial Resistance Surveillance Network (EARS-Net), more than 35,000 deaths annually in Europe are attributed to infections caused by antimicrobial-resistant microorganisms [3]. Several factors contribute to the dissemination of resistance in *Salmonella* spp., including selective pressure derived from excessive antimicrobial use in humans and animals, horizontal gene transfer mediated by plasmids, and the emergence of multidrug-resistant (MDR) strains [4,5]. Recent analysis of caecal samples from fattening pigs at slaughterhouses in 2023 revealed that 56.9% of *Salmonella* Typhimurium isolates and 81.1% of its monophasic variant were MDR. Notably, high resistance levels have been reported against ampicillin, sulfamethoxazole, tetracycline, and chloramphenicol, corresponding to the characteristic MDR resistance profile in *Salmonella* [4].

The excessive use of antimicrobials in food producing animals imposes a selective pressure that promotes the enrichment of resistant bacterial populations and favors the persistence and transmission of antimicrobial resistance genes. The One Health approach recognizes reducing antimicrobial use as a key strategy to mitigate selective pressure and limit the emergence and dissemination of antimicrobial-resistant bacteria across interconnected animal, human and environmental settings [6].

Among the antimicrobials most commonly used in food-producing animals, tetracyclines are broad-spectrum antibacterial agents with relatively low toxicity that have been widely used in both human and veterinary medicine, as well as historically as growth promoters in animal production. This extensive use has contributed to the emergence and dissemination of resistance [7,8]. Tetracycline resistance is mainly mediated by *tet* genes encoding efflux pumps that reduce intracellular antibiotic accumulation. Several *tet* genes, including *tetA*, *tetB*, *tetC*, *tetD* and *tetG,* have been identified in different *Salmonella* serotypes. Among them, *tetA* and *tetB* are the most frequently reported tetracycline resistance genes in *Salmonella* isolates from food-producing animals, including swine. The frequent association of these genes with mobile genetic elements, such as plasmids and transposons, contributes to their horizontal dissemination among bacterial populations, highlighting their relevance in the spread of tetracycline resistance [8]. In addition, *tetA* and *tetB* were detected among multidrug-resistant *Salmonella* Typhimurium and its monophasic variant isolated from pigs in southern Spain, supporting their selection as molecular targets in the present study [9].

As conventional antibiotics lose efficacy, plant-derived secondary metabolites, particularly essential oils (EOs), have emerged as promising candidates to mitigate AMR [10]. EOs are complex mixtures of volatile, lipophilic compounds, generally dominated by a few major constituents that can account for 20–70% of their composition. Their biological activity is largely attributed to these major components, which are mainly responsible for their antimicrobial effects [11,12].

Carvacrol (C_10_H_14_O) is a phenolic monoterpenoid predominantly found in the essential oils of oregano (*Origanum vulgare*) and thyme (*Thymus vulgaris*) [13]. It exerts broad-spectrum antimicrobial activity against clinically relevant pathogens of public health importance such as *Staphylococcus aureus*, *Klebsiella pneumoniae*, *Escherichia coli*, *Listeria monocytogenes* and *Salmonella enterica* [14,15]. In addition, carvacrol has gained increasing attention in the food industry as an antimicrobial and antioxidant food additive and in the development of antimicrobial packaging systems [16].

Previous studies evaluating the minimum inhibitory concentration (MIC) and minimum bactericidal concentration (MBC) have demonstrated that carvacrol inhibits the growth of different *Salmonella* spp. strains, although its efficacy appears to be strain-dependent [17,18,19,20].

In this context, natural compounds such as carvacrol can be proposed as antibiotic adjuvants capable of enhancing antimicrobial efficacy and counteracting resistance mechanisms, such as efflux pump activity and membrane permeability alterations. Although synergistic interactions between carvacrol and conventional antimicrobials have been reported in several bacterial species, most studies have focused exclusively on growth inhibition assays. More recently, proteomic approaches have been employed to investigate the molecular response of *Salmonella* to natural compounds, including carvacrol, providing new insights into their mechanisms of action. These studies identified changes in the expression of proteins involved in biofilm formation, oxidative stress response, cellular protein synthesis and DNA synthesis, highlighting that the antibacterial activity of these compounds in-volves multiple cellular targets beyond membrane disruption alone [21,22]. However, despite these advances, the effect of carvacrol on the expression of specific antimicrobial resistance genes remains poorly understood, particularly in MDR *Salmonella* of swine origin [23,24].

Despite the documented phenotypic synergy of carvacrol, its molecular impact on specific tetracycline resistance determinants (*tet* genes) in MDR swine isolates remains largely unexplored. Therefore, this study aimed to investigate the antimicrobial interaction between carvacrol and tetracycline and to assess the modulatory effect of carvacrol on the expression of tetracycline resistance-associated genes (*tetA* and *tetB*) in swine-derived MDR *Salmonella* Typhimurium.

## 2. Material and Methods

### 2.1. Samples and Antimicrobial Products

Seventy-three *Salmonella* Typhimurium isolates from the strain collection of the Animal Health Department (Faculty of Veterinary Medicine, University of Cordoba, Spain) were included in this study. The isolate collection has been previously described by Galán-Relaño et al. [9]. The isolates originated from several epidemiological studies conducted in different free-range pig farms and were collected between 2010 and 2018. Samples originated from multiple sources, including lymph nodes, faeces, tonsils, pens, transport, cutting and slaughter, and therefore each isolate was considered independent, as previously reported [9]. Antimicrobial susceptibility had previously been determined against seven antimicrobial categories, as described by Galán-Relaño et al. Multidrug resistance (MDR) was defined according to the criteria proposed by Magiorakos et al. [25] as acquired non-susceptibility to at least one antimicrobial agent in three or more antimicrobial categories. Accordingly, the 14 isolates selected for the checkerboard assays and the six isolates subsequently used for gene expression analyses fulfilled the MDR definition (Table S1).

Serotyping was performed by slide agglutination according to the Kauffmann–White–Le Minor scheme [26] and phage typing was conducted at the National Reference Laboratory for *Salmonella* and *Shigella* (Majadahonda, Spain) [9]. All isolates were stored at −20 °C in Brain Heart Infusion (BHI) broth (Oxoid^TM^) supplemented with 15% glycerol until use. Carvacrol (≥98% purity) and tetracycline (98–102% purity) were purchased from Sigma-Aldrich Laboratories (St. Louis, MO, USA). Tetracycline was selected as the antibiotic of interest due to the high prevalence of resistance reported in *Salmonella* spp. isolated from swine [4].

### 2.2. MIC and MBC Determination

All 73 isolates had been previously characterized as tetracycline-resistant [9]. The minimum inhibitory concentration (MIC) of carvacrol and tetracycline against *Salmonella* Typhimurium was determined by broth microdilution following the Clinical and Laboratory Standards Institute (CLSI) guidelines for bacteria isolated from animals [27]. While carvacrol MIC was evaluated for all 73 isolates, tetracycline MIC was re-evaluated in 14 representative strains using an expanded concentration range to determine the exact MIC values required for subsequent checkerboard assays. These 14 strains were selected to represent the phenotypic and genotypic diversity observed within the collection, including different phagetypes, carvacrol susceptibility profiles, tetracycline resistance phenotypes, and the presence of the *tetA* and *tetB* genes. The characteristics of the selected isolates are summarized in Supplementary Table S1.

Stock solutions were prepared at concentrations of 128 mg/mL for carvacrol and 8192 µg/mL for tetracycline. Carvacrol was dissolved in sterile distilled water containing 0.1% dimethyl sulfoxide (DMSO), whereas tetracycline was dissolved in sterile distilled water. Carvacrol stock solutions were vortex-mixed vigorously before use and immediately before dispensing into each individual well during the preparation of the serial dilutions to ensure homogeneous dispersion. Prior to the antimicrobial susceptibility assays, the effect of 0.1% DMSO on bacterial growth was evaluated by incubating the study isolates in the presence of 0.1% DMSO alone, and no inhibitory effect was observed. Twofold serial dilutions were prepared in 96-well microplates to obtain the desired concentration ranges.

Bacterial inocula were prepared from overnight cultures on Tryptic Soy Agar (TSA; Oxoid) and adjusted to an optical density equivalent to approximately 10^8^ CFU/mL. A working bacterial suspension of approximately 1 × 10^6^ CFU/mL was prepared. Equal volumes of this suspension and the antimicrobial solution were dispensed into each well, resulting in a final inoculum of approximately 5 × 10^5^ CFU/mL, in accordance with CLSI guidelines. Following inoculation, plates were incubated at 37 °C for 18–24 h. MIC, MIC_50_, and MIC_90_ values were determined. MIC endpoints were determined by visual inspection and defined as the lowest antimicrobial concentration showing complete absence of visible bacterial growth, in accordance with CLSI guidelines. Any well showing visible bacterial growth, including partial or trailing growth, was considered positive for bacterial growth and was therefore not considered as an inhibitory endpoint. Quality control was performed using *Escherichia coli* ATCC 25922, and the tetracycline MIC obtained for the quality control strain was within the CLSI-recommended quality control range.

Minimum bactericidal concentration (MBC) was determined only for carvacrol, as the primary objective was to evaluate the bactericidal activity of the natural compound under investigation. For MBC determination, 100 µL aliquots from the MIC well and the two higher antimicrobial concentrations were subcultured onto TSA plates and incubated at 37 °C for 24 h. The MBC was defined as the lowest antimicrobial concentration yielding no bacterial growth after subculture, corresponding to at least a 99.9% reduction in the initial bacterial inoculum.

All MIC and MBC determinations were performed in duplicate. When duplicate determinations yielded identical values, the result was accepted. If duplicate determinations differed, the assay was repeated in triplicate, and the modal MIC value was recorded as the final result.

### 2.3. Antimicrobial Interaction Test

The combined effect of carvacrol and tetracycline was evaluated in 14 representative tetracycline-resistant *Salmonella* Typhimurium strains using the checkerboard method as previously described [23]. The characteristics of the selected isolates are summarized in Supplementary Table S1.

Briefly, twofold serial dilutions of tetracycline (11 concentrations) and carvacrol (7 concentrations) were prepared in 96-well microplates. Combinations were tested starting from concentrations equivalent to 4 × MIC of each compound for the corresponding strain. Each well contained 100 µL of standardized bacterial inoculum and appropriate volumes of both antimicrobial agents. Positive (growth) and negative (sterility) controls were also included, and plates were incubated at 37 °C for 24 h under aerobic conditions.

The Fractional Inhibitory Concentration Index (FICI) was calculated for each antimicrobial combination using Microsoft Excel (Microsoft Corporation, Redmond, WA, USA) according to the following formula:

$${FICI}_{index}={FIC}_{CAR}+{FIC}_{TET}$$

$${FIC}_{CAR}=\frac{{MIC}_{CAR}\;in\;combination}{{MIC}_{CAR}}$$

$${FIC}_{TET}=\frac{{MIC}_{TET}\;in\;combination}{{MIC}_{TET}}$$

where [CAR] and [TET] represent the concentrations of carvacrol and tetracycline in combination that inhibited bacterial growth, whereas MIC_CAR_ and MIC_TET_ correspond to the MIC values of each compound when used alone. Interpretation of FICI values followed the European Committee on Antimicrobial Susceptibility Testing (EUCAST) criteria [28]: synergistic (FICI ≤ 0.5), additive (0.5 < FICI ≤ 1), indifferent (1 < FICI ≤ 2), or antagonistic (FICI > 2). For each strain, FICI values were calculated for all inhibitory wells showing complete absence of visible bacterial growth, and the interaction classification was based on the lowest FICI value obtained (best inhibitory combination), as has been previously reported in checkerboard assays [29,30]. All checkerboard assays were performed in duplicate.

MIC values used for FIC/FICI calculations were obtained from the control row and column of the corresponding checkerboard assay rather than from the MIC values obtained in the independent broth microdilution assay described in Section 2.2, in order to minimize inter-assay variability, as previously recommended [31,32]. Minor differences between MIC values obtained in independent broth microdilution assays and those determined within checkerboard assays are expected because of normal assay variability, with reproducibility generally within one two-fold dilution [27,33].

### 2.4. Growth Curve Test

Growth curve assays were performed to determine the subinhibitory concentrations of carvacrol, tetracycline, and their combination for subsequent gene expression analysis. Several subinhibitory concentrations derived from checkerboard assays were initially evaluated, and the final conditions (Table 1) were selected to allow all isolates to reach a comparable mid-exponential growth phase before RNA extraction, thereby minimizing differences associated with growth stage during subsequent gene expression analyses. The selected concentrations corresponded to approximately 0.015× to 0.062× MIC, depending on the isolate and treatment, and were chosen based on their ability to permit bacterial growth to the mid-exponential phase without complete growth inhibition. For each isolate, the concentrations selected for the combination treatment were also evaluated individually for carvacrol and tetracycline to allow direct comparison with the combined treatment. Growth curves of the six selected *Salmonella* Typhimurium isolates exposed to the selected subinhibitory concentrations are presented in Supplementary Figure S1. The assay was conducted over 24 h following the methodology described by Giovagnoni et al. [34].

Six strains were selected to represent the different interaction profiles identified in the checkerboard assay (synergistic, additive, and indifferent), allowing evaluation of gene expression responses across the range of phenotypic interactions observed. Bacterial cultures were prepared from overnight growth on tryptic soy agar (TSA, Oxoid) and inoculated into Mueller–Hinton broth (MHB) to an initial optical density (OD_595_) of 0.05. Cultures were incubated at 37 °C under aerobic conditions in the presence of carvacrol, tetracycline, or their combination at the selected concentrations. A control without antimicrobial agents was included. Bacterial growth was monitored by measuring optical density (OD_595_) at 1-h intervals until the stationary phase was reached, with a final measurement at 24 h, using a Multiskan FC spectrophotometer (Thermo Scientific). Each experimental condition was performed in duplicate.

### 2.5. RNA Extraction and cDNA Synthesis

Bacterial samples were collected during the mid-exponential growth phase for each experimental condition, as determined by the growth curve assay. Cells were harvested by centrifugation at 4000 rpm for 20 min at 4 °C, washed three times with phosphate-buffered saline (PBS), and resuspended in PBS. The resulting pellets were stored at −80 °C until further processing. For cell lysis, bacterial pellets were resuspended in TRI Reagent^®^ and mechanically disrupted using a combination of 0.1 mm and 0.5 mm silica beads (Benchmark Scientific^®^, Sayreville, NJ, USA) in a bead-beating system (Genie™, CViral, Spain). The disruption protocol consisted of four cycles of 1 min each, with intermediate cooling on ice. Lysates were clarified by centrifugation at 15,000 rpm for 5 min, and supernatants were collected and stored at −20 °C until RNA extraction.

Total RNA was extracted using the Direct-zol™ RNA Miniprep Plus kit (Zymo Research, Irvine, CA, USA) according to the manufacturer’s instructions. RNA concentration and purity were determined using a NanoDrop spectrophotometer (Thermo Fisher Scientific, Waltham, MA, USA) by measuring the A260/280 and A260/230 absorbance ratios. Only RNA samples showing purity values within the accepted ranges (A260/280 and A260/230 ratios between 1.8 and 2.2) were used for cDNA synthesis. To remove residual genomic DNA, samples were treated with the DNA-free™ Kit (Invitrogen, Carlsbad, CA, USA) following the manufacturer’s instructions. Purified RNA preparations were stored in RNase-free tubes at −80 °C.

Complementary DNA (cDNA) synthesis was performed using SuperScript™ II Reverse Transcriptase (Invitrogen) following the manufacturer’s instructions. The resulting cDNA was purified using the ZR-96 ChIP DNA Clean & Concentrator kit (Zymo Research) and stored at −20 °C until further analysis.

### 2.6. Quantitative Real-Time PCR (qPCR)

Quantitative real-time PCR (qPCR) was performed to evaluate the expression of tetracycline resistance genes. This analysis was designed as an exploratory assessment of transcriptional responses across multiple clinical isolates representing different interaction phenotypes (synergistic, additive, and indifferent), with the aim of identifying potential molecular patterns and generating hypotheses for future studies. Accordingly, each isolate and treatment condition was analysed from a single biological culture.

Five candidate reference genes (*16S rRNA*, *icdA*, *rpoD*, *gapA*, and *gmk*) were initially assessed for transcript stability, and the most stable genes were selected for normalization. The target genes analysed were *tetA* and *tetB*. Primer sequences and their respective melting temperatures (Tm) are listed in Table 2.

Reactions were carried out using the iTaq™ Universal SYBR^®^ Green Supermix (Bio-Rad Laboratories, Hercules, CA, USA) at a final volume of 10 µL, containing 5 µL of SYBR Green Supermix, 0.5 µL of each primer (10 µM), 2 µL of cDNA (1 ng/µL), and 2 µL nuclease-free water. All qPCR reactions were performed in technical triplicate, and mean Ct values were used for subsequent analyses. Assays were performed using a CFX Duet Real-Time PCR System (Bio-Rad Laboratories, Hercules, CA, USA), and data were analysed using CFX Maestro software version 2.3 (Bio-Rad Laboratories, Hercules, CA, USA).

The amplification protocol consisted of an initial denaturation step at 95 °C for 3 min, followed by 40 cycles of 95 °C for 15 s and annealing/extension at the specific primer melting temperature (Tm) for 30 s (Table 2). A melting curve analysis (65–95 °C) was performed at the end of each run to confirm amplification specificity.

To ensure the absence of genomic DNA contamination and reagent impurities, negative controls including no-template controls (NTC) and no-reverse transcriptase (no-RT) controls were included in each run. No amplification was detected in any negative control.

### 2.7. Relative Gene Expression Analysis

Relative gene expression was calculated using the Pfaffl method [40], which accounts for differences in amplification efficiency between target and reference genes.

Primer efficiency (E) was determined from standard curves generated using serial tenfold dilutions of *Salmonella* genomic DNA (50 to 0.005 ng/µL) extracted using the QIAamp DNA kit (Qiagen).

Efficiency for each primer pair was calculated from the slope (m) of the standard curve using the equation E = 10^−1/m^. Efficiency was additionally expressed as a percentage: E (%) = (10^(−1/m)^ − 1) × 100. Only primer pairs with efficiencies between 90 and 110% were considered acceptable or ideal.

The stability of candidate reference genes (*16S rRNA*, *icdA*, *rpoD*, *gapA*, and *gmk*) was evaluated across all experimental conditions. Following the principles proposed by Vandesompele et al. [41], for the use of multiple stable reference genes in qPCR normalization, the variability of Ct values across isolates and experimental conditions was assessed for each candidate reference gene. The two genes showing the lowest Ct variability (*rpoD* and *gmk*) were selected and used for multigene normalization.

Relative expression ratios were calculated using a modified Pfaffl model with multiple reference genes, using the geometric mean of the reference genes as follows:

$$Ratio=\;\frac{E_{gen}^{\Delta Ct(control-sample)}}{\sqrt[{2}]{E_{ref1}^{\Delta Ct-ref1\left(control-sample\right)\;\times \;}E_{ref2}^{\Delta Ct-ref2\left(control-sample\right)}}}$$

The control condition corresponded to bacteria grown in MHB without antimicrobial agents, whereas treated samples included exposure to carvacrol, tetracycline, or their combination.

To specifically assess the modulatory effect of carvacrol during combined exposure, relative expression of *tetA* and *tetB* was additionally calculated using the tetracycline-only treatment as calibrator (CA + TET/TET). Ratios were log_2_-transformed [log_2_(CA + TET/TET)] for interpretation, where negative values indicate downregulation and positive values indicate upregulation relative to tetracycline treatment.

### 2.8. Statistical Analysis

Statistical analyses were performed using GraphPad Prism version 8.0.1 (GraphPad Software, San Diego, CA, USA). MIC, MBC, checkerboard, and growth curve data were analysed descriptively. Gene expression data were also primarily analysed descriptively because no independent biological replicates were available for each isolate and treatment condition. Owing to the exploratory nature of the gene expression analysis, the limited sample size, and the absence of independent biological replicates, statistical power analyses, confidence intervals, and effect size measures were not considered appropriate. Paired comparisons between *tetA* and *tetB* expression were evaluated using the Wilcoxon matched-pairs signed-rank test. Differences were considered statistically significant at *p* ≤ 0.05. An exploratory Spearman rank correlation analysis was performed to assess the association between tetracycline MIC and carvacrol MIC. The analysis was initially performed using the 14 isolates with quantitative tetracycline MIC values. A second exploratory analysis included all 73 isolates by assigning a value of 128 µg/mL to isolates reported as having tetracycline MIC values ≥64 µg/mL, thereby enabling rank-based comparison.

## 3. Results

### 3.1. Antimicrobial Susceptibility of Salmonella Typhimurium Isolates to Carvacrol and Tetracycline

**Figure 1:**
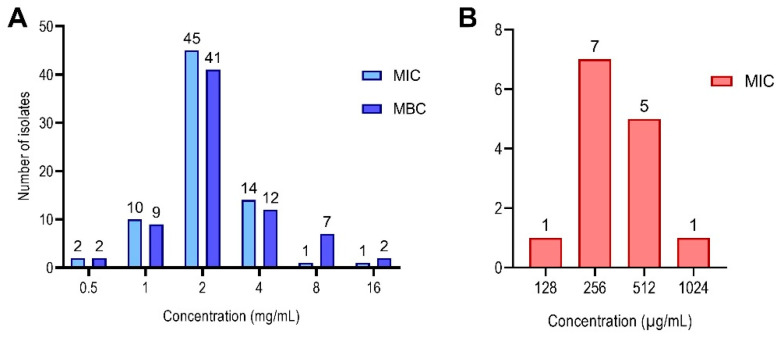
Distribution of antimicrobial susceptibility values for carvacrol and tetracycline. (**A**) Minimum inhibitory concentration (MIC) and minimum bactericidal concentration (MBC) of carvacrol against 73 *Salmonella* Typhimurium isolates. (**B**) MIC values of tetracycline against 14 selected tetracycline-resistant *Salmonella* Typhimurium isolates, determined independently by broth microdilution as described in Section 2.2. Values are expressed in mg/mL for carvacrol and µg/mL for tetracycline.

For carvacrol, MIC values against the 73 *Salmonella* strains ranged from 0.5 to 16 mg/mL. The most frequent MIC value was 2 mg/mL (61.6%), followed by 4 mg/mL (19.2%) and 1 mg/mL (13.7%) (Figure 1A). The MIC_50_ and MIC_90_ values were 2 mg/mL and 4 mg/mL, respectively. MBC values coincided with their corresponding MICs in 84.9% of the isolates, suggesting a predominantly bactericidal effect. The most frequent MBC value was 2 mg/mL, followed by 4 mg/mL and 1 mg/mL (Figure 1A). The MBC_50_ and MBC_90_ values were 2 mg/mL and 8 mg/mL, respectively.

Regarding tetracycline, more homogeneous MIC values were observed among the 14 selected isolates. Seven isolates (50%) exhibited an MIC of 256 µg/mL, representing the MIC_50_ value, whereas five isolates showed an MIC of 512 µg/mL, corresponding to the MIC_90_ value (Figure 1B).

An exploratory Spearman rank correlation analysis was performed to evaluate the relationship between tetracycline MIC and carvacrol MIC. First, the analysis was conducted using the 14 isolates for which quantitative tetracycline MIC values were available, and no statistically significant correlation was observed (Spearman’s ρ = −0.492, *p* = 0.099). A second exploratory analysis including all 73 isolates was then performed by assigning a value of 128 µg/mL to tetracycline MIC values reported as ≥64 µg/mL to enable rank-based comparison. Likewise, no significant correlation was detected (Spearman’s ρ = −0.1589, *p* = 0.179). These findings suggest that tetracycline resistance levels were not associated with reduced susceptibility to carvacrol among the isolates evaluated.

### 3.2. Interaction Between Carvacrol and Tetracycline

The interaction between carvacrol and tetracycline varied among isolates, revealing strain-dependent antimicrobial responses in the 14 *Salmonella* Typhimurium isolates tested. Synergistic activity (FICI ≤ 0.5) was observed in 14.3% (*n* = 2) of the isolates, using combinations of 2 mg/mL carvacrol with 32 or 64 µg/mL tetracycline (Table 3). In these isolates, the combination reduced the inhibitory concentration of carvacrol fourfold (from 8 to 2 mg/mL) and that of tetracycline fourfold compared with the MICs of each antimicrobial tested alone.

Additionally, additive interactions were detected in 50.0% (*n* = 7) of the isolates, while 28.6% (*n* = 4) showed indifferent responses. Antagonism was observed in one isolate (7.1%) (Table 3).

To further quantify the impact of the combination on tetracycline activity, MIC reduction was expressed as the fold reduction relative to tetracycline used alone. Synergistic isolates showed the highest reduction, with a median of 4-fold (IQR: 4–4). Additive isolates exhibited a moderate reduction (median: 2-fold; IQR: 2–2), whereas indifferent isolates showed almost no reduction (median: 1-fold; IQR: 1–1). These findings are consistent with the interaction patterns observed in the checkerboard assay.

### 3.3. Effect on Resistance Gene Expression

Growth curve assays confirmed that the selected subinhibitory concentrations caused only moderate reductions in bacterial growth compared with untreated controls and did not prevent progression to the exponential phase. Consequently, samples for gene expression analysis were collected at the mid-exponential phase. Candidate reference genes exhibited variable expression stability across strains and treatment conditions; therefore, *rpoD* and *gmk* were selected as the most stable reference genes for combined normalization of qPCR data.

**Figure 2:**
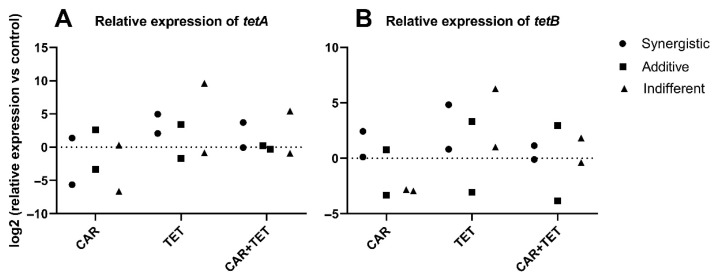
Relative expression of *tetA* (**A**) and *tetB* (**B**) in individual *Salmonella* Typhimurium isolates under different treatment conditions. Data are expressed as log2 fold change relative to the untreated control (baseline = 0). Each symbol represents an individual isolate, categorized by its interaction phenotype: synergistic (circles), additive (squares), and indifferent (triangles). CAR: carvacrol; TET: tetracycline.

Under single-compound exposure, gene expression responses varied among isolates. Carvacrol alone generally produced lower or more moderate expression changes in *tetA* and *tetB*, whereas tetracycline treatment more frequently resulted in higher expression levels, particularly for *tetA*. Gene expression profiles across all treatment conditions are summarized in Figure 2.

**Figure 3:**
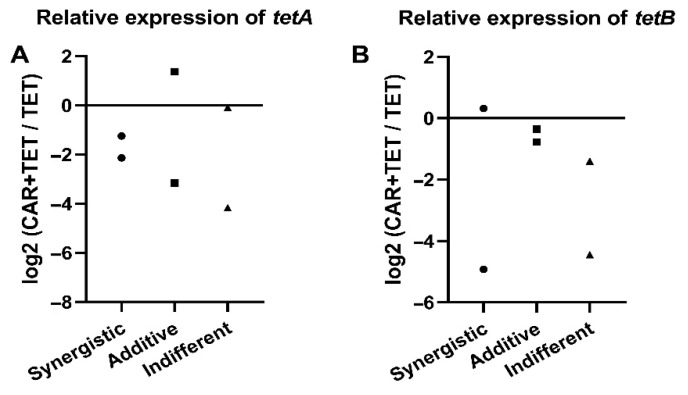
Modulatory effect of carvacrol on *tetA* (**A**) and *tetB* (**B**) expression during combined treatment. Data are expressed as log2 fold change in the combination (CAR + TET) relative to tetracycline (TET) alone. Each symbol represents an individual *Salmonella* Typhimurium isolate grouped according to its interaction phenotype (synergistic, additive, or indifferent). Negative values indicate that the addition of carvacrol reduced gene expression compared with tetracycline alone. CAR, carvacrol; TET, tetracycline.

To assess the effect of carvacrol on the tetracycline response, gene expression under combined treatment was compared with tetracycline exposure alone (Figure 3). For *tetA*, relative expression under combined treatment was reduced in five of the six evaluated isolates (83.3%), with expression ratios ranging from 0.056- to 0.946-fold. Both isolates previously classified as synergistic showed decreased expression, whereas additive and indifferent isolates displayed more heterogeneous responses, including one isolate with increased expression (2.581-fold).

Regarding *tetB*, relative expression was also reduced in 83.3% (*n* = 5) of the isolates, with ratios ranging from 0.033- to 0.781-fold. One synergistic isolate showed marked repression (0.033-fold), whereas the remaining responses varied from moderate reduction to expression levels close to the tetracycline control.

When stratified by interaction phenotype, all synergistic and indifferent isolates exhibited downregulation of *tetA*. In contrast, *tetB* showed more variable responses in the synergistic group but consistent downregulation in additive and indifferent isolates. Overall, downregulation under combined treatment was observed in most of the isolates for both genes.

**Figure 4:**
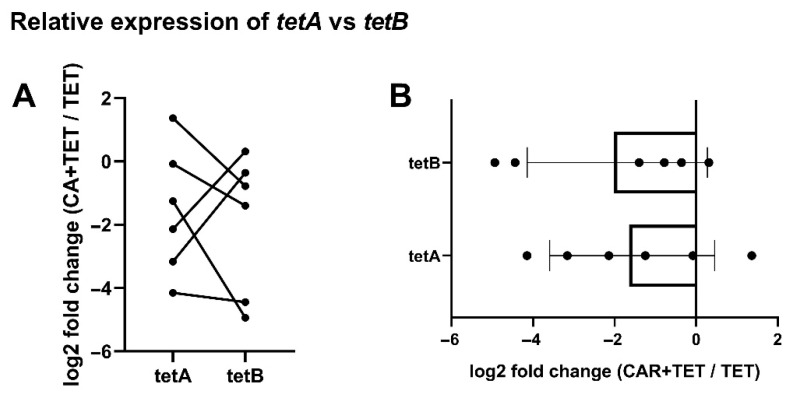
Comparative analysis of *tetA* and *tetB* modulation under combined treatment. Data represents the log_2_ fold change in the combination (CAR + TET) relative to tetracycline (TET) alone. (**A**) Paired comparison of gene expression within individual isolates; lines connect *tetA* and *tetB* values for the same isolate to illustrate differential regulation patterns. (**B**) Distribution of relative expression values across all isolates; each point represents an individual isolate, and box plots indicate the median and interquartile range (IQR). No significant differences were detected between the expression levels of the two genes (*p* > 0.05).

A paired comparison of gene expression under combined treatment revealed isolate-specific differences between *tetA* and *tetB* (Figure 4). Although both genes generally showed reduced expression relative to tetracycline alone, the magnitude and direction of the response varied between isolates, and no consistent pattern of differential regulation between *tetA* and *tetB* was detected (*p* > 0.05). Taken together, these findings indicate that the combined treatment was associated with heterogeneous, but predominantly repressive, transcriptional responses of tetracycline resistance-associated genes across the isolates evaluated.

## 4. Discussion

The increasing dissemination of antimicrobial resistance (AMR), particularly among multidrug-resistant (MDR) *Salmonella* Typhimurium strains of swine origin, represents an important challenge for both public and animal health, as resistance increasingly compromises the efficacy of conventional antimicrobials, including tetracyclines [42,43].

In the present study, the antimicrobial effect of carvacrol was evaluated against 73 swine-derived *Salmonella* Typhimurium strains. MIC values ranged from 0.5 to 16 mg/mL, with variability observed among strains. MBC values coincided with the MIC values in most isolates, suggesting a predominantly bactericidal effect, consistent with the results described by Berdejo et al. [44] and Trevisan et al. [20]. Similar findings have also been reported in recent studies describing the antimicrobial efficacy of carvacrol-rich essential oils against *Salmonella* isolates from the swine food chain [45,46]. However, the concentrations observed in the present study were higher than those commonly reported in previous studies, in which the MIC of carvacrol against *Salmonella* Typhimurium has typically ranged from 0.064 to 0.6 mg/mL [17,20,24,44,47,48]. Comparable carvacrol concentrations have nevertheless been reported against pathogens with high physiological tolerance including *Pseudomonas aeruginosa* and multidrug-resistant *Acinetobacter baumannii* [49,50]. Cho and Lee [51] reported MIC values of 15 and 16 mg/mL against *Staphylococcus aureus* and *Escherichia coli* O157:H7 strains in milk, respectively, likely reflecting reduced carvacrol bioavailability in a lipid-rich food matrix rather than a methodological discrepancy. These findings suggest that carvacrol susceptibility may vary among bacterial populations, resulting in different MIC values across studies. To investigate whether this variability could be associated with the degree of tetracycline resistance, an exploratory correlation analysis was performed. Exploratory correlation analyses did not reveal a significant association between tetracycline MIC and carvacrol susceptibility, suggesting that tetracycline resistance level was not predictive of reduced susceptibility to carvacrol in the isolates evaluated. Our results contrast with those of Fernández-Márquez et al., who reported a positive correlation between tetracycline resistance and carvacrol tolerance in *Salmonella* isolates from hen eggshells [52]. However, they are consistent with the observations of Ravishankar et al., who demonstrated that carvacrol effectively inactivated both antibiotic-resistant and antibiotic-susceptible *Salmonella enterica* isolates, indicating that conventional antibiotic resistance does not necessarily compromise susceptibility to carvacrol [53]. Differences in isolate origin, epidemiological background, antimicrobial exposure history, and study design may explain these contrasting observations.

In addition to strain variability, MIC values may also be influenced by experimental conditions and the physicochemical properties of carvacrol. Factors such as pH, medium composition, and solvent selection may affect its antimicrobial activity, bioavailability, and interaction with bacterial membranes [54,55]. In the present study, 0.1% DMSO was used as a solubilizing agent, consistent with previous studies evaluating hydrophobic compounds such as carvacrol [17,56].

Furthermore, the selected isolates exhibited high tetracycline resistance levels (MIC_90_ = 512 µg/mL), highlighting the need to identify compounds capable of enhancing tetracycline efficacy against resistant *Salmonella* strains.

The combination of carvacrol and tetracycline showed strain-dependent interactions, ranging from synergistic and additive to indifferent and antagonistic responses. In strains where synergistic interactions were observed, up to a fourfold reduction in the effective concentration of tetracycline was achieved. Similar findings have been reported for tetracycline-based combinations. Palaniappan and Holley [57] demonstrated synergistic interactions between carvacrol and tetracycline against *Salmonella* Typhimurium and *Escherichia coli*, whereas Huerta Lorenzo et al. [58] reported predominantly additive effects between *Origanum vulgare* essential oil (containing 63.01% carvacrol) and oxytetracycline in multidrug-resistant *Salmonella enterica* isolates. More broadly, carvacrol has also been shown to enhance the activity of different antibiotic classes [57,59]. Consistent with our findings, Solarte et al. [60] also reported strain-dependent responses in *Salmonella* Typhimurium, supporting the notion that the efficacy of antimicrobial combinations may vary according to both the isolate and the antimicrobial used.

One possible explanation for the synergistic and additive interactions observed in the present study is the ability of phenolic compounds such as carvacrol and thymol to alter membrane integrity and interfere with efflux pump activity, thereby increasing intracellular antibiotic accumulation and enhancing antimicrobial efficacy, as suggested in previous studies [59,61]. However, the interaction between carvacrol and tetracycline varied among isolates, ranging from synergistic to indifferent and antagonistic responses. Similar isolate-dependent variability was reported by Cirino et al. [62] in clinical isolates of *Acinetobacter baumannii*, where differential responses to carvacrol and thymol combinations were associated with distinct resistance profiles identified through genomic analysis. These findings underscore the importance of strain-specific factors when evaluating the efficacy of antimicrobial combinations against MDR pathogens.

Although the checkerboard assay is widely used for the initial evaluation of antimicrobial interactions, it should be considered a screening method and interpreted together with complementary approaches. In the present study, the checkerboard assay was used to characterize the interaction profiles of the different *Salmonella* Typhimurium isolates and to identify the most promising antimicrobial combinations. The selected combinations were subsequently evaluated by growth curve analysis and used to select the subinhibitory treatment conditions for the gene expression analyses.

Growth curve assays using subinhibitory concentrations of carvacrol, tetracycline, and their combination allowed moderate growth inhibition while maintaining progression to the exponential phase. Conditions causing complete growth inhibition or insufficient bacterial growth were excluded, as these may substantially alter transcriptional responses and compromise downstream gene expression analyses [63,64]. These assays were designed to standardize RNA sampling at a comparable mid-exponential growth phase among isolates, thereby minimizing differences associated with growth stage. However, subtle differences in bacterial growth kinetics under subinhibitory antimicrobial exposure cannot be completely excluded and may have influenced resistance gene expression. Further studies are warranted to investigate this relationship. Under the experimental conditions evaluated in the present study, gene expression analyses revealed differential responses depending on the antimicrobial treatment applied.

Exposure to tetracycline alone resulted in increased expression of both *tetA* and *tetB*, particularly the former. This finding is consistent with the role of tetracycline as an inducer of tetracycline resistance determinants, including efflux-associated *tet* genes. Among these determinants, *tetA* is one of the most frequently detected tetracycline resistance genes in *Salmonella* spp. [8].

The combination of tetracycline and carvacrol resulted in a decrease in the relative expression of *tetA* and *tetB* compared with tetracycline-only treatment in five of the six isolates. One possible explanation for this observation may be related to the reported mechanism of action of carvacrol. Due to its lipophilic nature, carvacrol has been shown to insert into the bacterial lipid bilayer, increasing membrane permeability, depolarizing the membrane, and dissipating the proton motive force (PMF) [65]. Since tetracycline efflux systems such as TetA and TetB depend on the transmembrane proton gradient for antibiotic extrusion, disruption of PMF has been hypothesized to interfere with efflux activity and promote increased intracellular accumulation of tetracycline [61,66,67]. If this mechanism operates under the conditions evaluated, the combined effect of carvacrol and tetracycline could enhance intracellular antibiotic retention and potentiate ribosomal inhibition [7], which, in turn, could contribute to the reduced expression of resistance-associated genes observed under combined exposure. However, these parameters were not evaluated in the present study, and therefore this mechanism should be regarded as a plausible hypothesis rather than a demonstrated explanation for the observed transcriptional changes. Beyond its direct effects on membrane integrity, carvacrol has also been reported to interfere with bacterial energy metabolism, resulting in ATP depletion and disruption of membrane-associated processes [65,66,67]. These physiological alterations may indirectly influence global regulatory networks involved in antimicrobial adaptation, including regulators controlling membrane homeostasis, oxidative stress responses, and multidrug efflux systems [68,69,70,71]. Although the present study focused exclusively on the expression of the tetracycline resistance determinants *tetA* and *tetB*, it is conceivable that the transcriptional changes observed under combined exposure also reflect broader regulatory responses rather than direct effects on these genes alone. Future transcriptomic and proteomic analyses will be necessary to determine whether global regulators and additional resistance-associated pathways contribute to the potentiating effect of carvacrol.

Overall, reduced expression of *tetA* and *tetB* was observed in 83.3% of the isolates under combined exposure, with repression ratios as low as 0.056-fold in some strains. Although previous studies have mainly focused on the modulation of non-specific efflux systems such as AcrAB-TolC [68,69], the present findings raise the possibility that carvacrol may influence the expression of tetracycline-specific resistance determinants in some isolates. Notably, the consistent downregulation observed for both genes may reflect a coordinated transcriptional response rather than a gene-specific effect, potentially reflecting indirect modulation of broader stress-response pathways involved in antimicrobial adaptation and efflux regulation [70,71].

The results of the present study are consistent with previous reports describing resistance gene modulation following exposure to carvacrol or related phenolic compounds. Shen et al. [72] demonstrated that carvacrol combined with streptomycin enhanced susceptibility in antibiotic-resistant strains through the downregulation of resistance-associated genes. Similarly, Bonetti et al. [73] reported reduced expression of resistance determinants following combined treatment with thymol and antibiotics in field strains of *Escherichia coli*. Beyond antimicrobial resistance, carvacrol has also been associated with altered expression of genes involved in quorum sensing [56,74], motility [72], virulence [34], and biofilm formation [75,76], supporting its broader modulatory effect on bacterial physiology.

However, resistance gene expression varied among strains, particularly in isolates exhibiting additive or indifferent interactions. Such heterogeneity is common in *Salmonella* populations and may reflect genomic plasticity, variability in resistance-associated regulatory networks, and adaptive responses to antimicrobial stress [71,77]. In *Salmonella*, resistance determinants such as *tetA* and *tetB* are frequently associated with plasmids, transposons, and diverse regulatory elements, which may influence both basal and inducible expression levels [78]. Moreover, Tet efflux systems are regulated through complex mechanisms involving TetR repressors and proton motive force-dependent transport.

Because tetracycline resistance determinants are embedded within broader regulatory networks, the observed transcriptional changes may also reflect indirect modulation of global stress-response pathways involved in membrane homeostasis and efflux regulation. Consequently, differences in membrane physiology, regulatory sequences, or intracellular tetracycline accumulation may substantially influence transcriptional responses among strains [42,78,79]. Previous exposure to antibiotics, disinfectants, or environmental stressors in animal production systems may further contribute to the selection of strains with distinct metabolic and stress-adaptation strategies, resulting in isolate-dependent responses under combined antimicrobial exposure [44,48].

Although subinhibitory concentrations varied among isolates, no consistent relationship between concentration level and gene expression changes was observed, suggesting that isolate-specific regulatory responses may play a more important role than concentration alone. Isolates previously classified as synergistic exhibited more consistent downregulation patterns, whereas additive and indifferent isolates showed greater variability, including one isolate with increased *tetA* expression. These findings suggest that modulation of *tet* gene expression may be associated with transcriptional response observed under combined exposure and could partially contribute to the observed phenotypic interaction; however antimicrobial activity is likely multifactorial and also involves membrane destabilization and other stress-associated mechanisms.

From a broader perspective, these findings are relevant within the One Health framework, which recognizes that reducing antimicrobial use in food-producing animals contributes to decreasing selective pressure and limiting the dissemination of antimicrobial-resistant bacteria across interconnected animal, human, and environmental settings [6]. If confirmed through further mechanistic and in vivo studies, the ability of carvacrol to enhance tetracycline activity and modulate the expression of resistance-associated genes could support the development of complementary strategies aimed at reducing antimicrobial use in swine production, thereby contributing to broader efforts to control the dissemination of antimicrobial resistance.

In line with the exploratory nature of this study, the gene expression analysis should be interpreted accordingly. RNA purity was verified before cDNA synthesis by measuring the A260/280 and A260/230 absorbance ratios. Likewise, RNA samples were treated with DNase I to remove residual genomic DNA, and no-RT controls were included in each qPCR run to confirm the absence of genomic DNA contamination; no amplification was observed in these controls, confirming successful removal of genomic DNA prior to cDNA synthesis. However, RNA integrity was not formally assessed using capillary electrophoresis or an equivalent method. This methodological limitation should be considered when interpreting the transcriptional data. The objective was not to establish statistically robust mechanistic relationships but to provide an initial assessment of transcriptional responses across multiple clinical isolates exhibiting different interaction phenotypes. Although no independent biological replicates were performed for each isolate, the inclusion of genetically distinct clinical isolates allowed the identification of transcriptional patterns across different strain backgrounds. Nevertheless, the limited sample size and the lack of independent biological replicates reduced the statistical power of the study and prevented robust inferential analyses. Consequently, these findings should be considered descriptive and hypothesis-generating, requiring confirmation in future studies incorporating a larger number of isolates, independent biological replication, and broader transcriptomic or proteomic approaches. Accordingly, the observed modulation of *tetA* and *tetB* expression should be interpreted as an exploratory finding rather than evidence of a causal mechanism underlying the interaction between carvacrol and tetracycline.

## 5. Conclusions

Overall, the present findings demonstrate that carvacrol exhibits in vitro antimicrobial activity and may enhance tetracycline activity in a subset of multidrug-resistant *Salmonella* Typhimurium isolates under the experimental conditions evaluated. Carvacrol exhibited intrinsic antimicrobial activity and, in combination with tetracycline, was associated with reduced *tetA* and *tetB* expression in most of the selected isolates. Nevertheless, the variability observed among strains highlights the importance of considering isolate-dependent responses when evaluating antimicrobial combinations. These findings support further mechanistic investigation and in vivo studies to evaluate carvacrol as a potential antimicrobial adjuvant for the control of swine-associated *Salmonella* infections. If confirmed under field conditions, this strategy could contribute to the development of complementary approaches aimed at optimizing antimicrobial use in swine production. The development of natural alternatives capable of enhancing the efficacy of conventional antibiotics may help reduce the selective pressure associated with antimicrobial resistance, consistent with the One Health approach to antimicrobial resistance control. Future research should also determine whether similar potentiating effects can be achieved with other classes of antimicrobials or complementary therapeutic strategies and should evaluate these approaches under in vivo conditions, while integrating transcriptomic, proteomic and other functional approaches to better characterize the molecular mechanisms underlying these interactions. Such studies will be essential to establish the translational potential of carvacrol as part of integrated strategies aimed at optimizing antimicrobial use in food-producing animals while limiting the emergence and dissemination of antimicrobial resistance.

## Figures and Tables

**Table 1:** Experimental conditions used for RNA extraction and qPCR analysis, including selected strains, interaction profiles, treatment concentrations, culture collection optical density (OD_595_), and exposure times.

Strain	Interaction Profile	CAR			TET			CAR + TET		
		Selected Concentration	OD_595_ at Harvest	Time (h)	Selected Concentration	OD_595_ at Harvest	Time (h)	Selected Concentration	OD_595_ at Harvest	Time (h)
3	S	0.015 × MIC	0.25	3.0	0.015 × MIC	0.25	3.0	0.015 × MIC_CAR_ + 0.015 × MIC_TET_	0.17	3.0
7	S	0.031 × MIC	0.22	3.0	0.031 × MIC	0.25	3.0	0.031 × MIC_CAR_ + 0.031 × MIC_TET_	0.24	2.0
1	A	0.015 × MIC	0.18	2.0	0.015 × MIC	0.17	2.0	0.015 × MIC_CAR_ + 0.015 × MIC_TET_	0.15	2.0
13	A	0.015 × MIC	0.17	2.0	0.015 × MIC	0.19	2.0	0.015 × MIC_CAR_ + 0.015 × MIC_TET_	0.14	2.0
6	I	0.031 × MIC	0.24	2.0	0.015 × MIC	0.28	3.0	0.031 × MIC_CAR_ + 0.015 × MIC_TET_	0.22	2.5
14	I	0.062 × MIC	0.24	2.0	0.062 × MIC	0.25	3.0	0.062 × MIC_CAR_ + 0.062 × MIC_TET_	0.15	3.5

Abbreviations: A: additivity; I: indifference; S: synergy; MIC: minimum inhibitory concentration; CAR: carvacrol; TET: tetracycline. Concentrations were selected from checkerboard-derived subinhibitory conditions that allowed bacterial growth to progress to the exponential phase without complete growth inhibition. Bacterial samples were harvested during the mid-exponential growth phase for each experimental condition.

**Table 2:** Primer sequences, melting temperatures (Tm), and amplification efficiencies of target and candidate reference genes evaluated for qPCR normalization.

Gene	Sequence (5′–3′)	Product Size (bp)	Tm	Amplification Efficiency (%)	References
*tetA-F*	GCTACATCCTGCTTGCCTTC	115	60 °C	94.01	[9]
*tetA-R*	*ACCTGCCTGGACAAAATTGC*				
*tetB-F*	CCGAAGTAGGGGTTGAGACG	103	60 °C	90.01	[9]
*tetB-R*	TGGCCTATCAATTGCGCTGA				
*16S rRNA-F*	AGGCCTTCGGGTTGTAAAGT	244	55 °C	98.03	[35]
*16S rRNA-R*	GACTCAAGCCTGCCAGTTTC				
*icdA-F*	TGGTATCGGTGTTGATGTCACTC	140	54 °C	93.34	[36]
*icdA-R*	CATCCTGGCCGTAAACCTGTGTG				
*rpoD-F*	GTGAAATGGGCACTGTTGAACTG	131	60 °C	99.89	[37]
*rpoD-R*	TTCCAGCAGATAGGTAATGGCTTC				
*gapA-F*	GGTGTTGACGTAGTGGCTGAA	204	60 °C	98.71	[38]
*gapA-R*	AGCGTTGGAAACGATGTCC				
*gmk-F*	TTGGCAGGGAGGCGT TT	84	60 °C	93.17	[39]
*gmk-R*	GCGCGAAGTGCCGTAGTAAT				

**Table 3:** Summary of checkerboard interaction outcomes between carvacrol and tetracycline against 14 multidrug-resistant *Salmonella* Typhimurium strains.

Interaction Outcome	FICI Interpretation	Number of Strains (*n* = 14)	FIC CAR (Range)	FIC TET (Range)	FICI (Range)
Synergistic	FICI ≤ 0.5	2 (14.3%)	0.25–0.25	0.25–0.25	0.5–0.5
Additive	0.5 < FICI ≤ 1	7 (50.0%)	0.125–0.5	0.0625–0.5	0.5625–1.0
Indifferent	1 < FICI ≤ 2	4 (28.6%)	0.125–1.0	0.25–1.0	1.125–1.5
Antagonistic	FICI > 2	1 (7.1%)	1.0	2.0	3.0

Abbreviations: CAR, carvacrol; TET, tetracycline; FICI, fractional inhibitory concentration index; FIC, fractional inhibitory concentration. The reported ranges correspond to the individual FIC values for carvacrol and tetracycline and the resulting FICI values within each interaction category.

## Data Availability

All data generated or analyzed during this study are included in this published article and its Supplementary Materials.

## Supplementary Materials

The following supporting information can be downloaded at: https://www.mdpi.com/article/10.3390/ani16152362/s1, Figure S1: Growth curves of selected *Salmonella* Typhimurium isolates cultured in Mueller–Hinton broth (MHB; control) or exposed to carvacrol (CAR), tetracycline (TET), and their combination (CAR+TET) at the selected subinhibitory concentrations; Table S1: Epidemiological, phenotypic and antimicrobial interaction characteristics of the 14 multidrug-resistant Salmonella Typhimurium isolates selected for checkerboard assays.
