# Supplementary material for: Carvacrol Modulates Tetracycline Susceptibility and Resistance Gene Expression in Multidrug-Resistant Salmonella Typhimurium Strains Isolated from Swine
Source: Animals (Basel). 2026 Aug 2;16(15):2362. doi: 10.3390/ani16152362 (PMC13463590; doi:10.3390/ani16152362)
Supplement: Supplementary file 1 [file animals-16-02362-s001.zip › Supplementary Table S1.pdf]

**Supplementary Table S1.** Epidemiological, phenotypic and antimicrobial interaction characteristics of the 14 multidrug-resistant *Salmonella* Typhimurium isolates selected for checkerboard assays.

| Strain | Phagotype | *Tet* genes | | Interaction | Lowest inhibitory combination | | MIC CAR (mg/mL) | MIC TET (µg/mL) | FIC CAR | FIC TET | FICI | Resistotype |
|---|---|---|---|---|---|---|---|---|---|---|---|---|
| | | *tetA* | *tetB* | | CAR (mg/mL) | TET (µg/mL) | | | | | | |
| **1** | **193** | **x** | **x** | **A** | **2** | **128** | **8** | **256** | **0.25** | **0.5** | **0.75** | **S-CF-TET-SXT-AMP-E-CHL** |
| 2 | 104b | x | x | I | 0.125 | 256 | 1 | 256 | 0.125 | 1 | 1.125 | S-CF-TET-AMP-E |
| **3** | **193** | **x** | **x** | **S** | **2** | **64** | **8** | **256** | **0.25** | **0.25** | **0.5** | **S-CF-TET-SXT-AMP-E-CHL** |
| 4 | 193 | x | x | I | 1 | 128 | 2 | 128 | 0.5 | 1 | 1.5 | S-CF-TET-SXT-AMP-E-CHL |
| 5 | U311 | x | x | A | 2 | 64 | 8 | 128 | 0.25 | 0.5 | 0.75 | S-CF-TET-AMP-CHL |
| **6** | **PNR** | **x** | **x** | **I** | **1** | **128** | **4** | **128** | **0.25** | **1** | **1.25** | **S-CF-TET-SXT-AMP-E-CHL** |
| **7** | **138** | **x** | **x** | **S** | **2** | **32** | **8** | **128** | **0.25** | **0.25** | **0.5** | **S-TET-SXT-AMP-E-CHL** |
| 8 | U311 | x | x | An | 1 | 512 | 1 | 256 | 1 | 2 | 3 | S-CF-TET-SXT-AMP-E-CHL |
| 9 | U311 | x | x | A | 0.25 | 128 | 1 | 256 | 0.25 | 0.5 | 0.75 | S-CF-TET-SXT-AMP-E-CHL |
| 10 | 193 | x | x | A | 2 | 256 | 4 | 512 | 0.5 | 0.5 | 1 | S-CF-TET-SXT-AMP-E-CHL |
| 11 | 193 | x | x | A | 0.5 | 128 | 4 | 256 | 0.125 | 0.5 | 0.625 | S-CF-TET-SXT-AMP-E-CHL |
| 12 | U311 | x | x | A | 0.5 | 32 | 1 | 512 | 0.5 | 0.0625 | 0.5625 | S-TET-AMP-E-CHL |
| **13** | **193** | **x** | **x** | **A** | **1** | **128** | **4** | **256** | **0.25** | **0.5** | **0.75** | **S-CF-TET-SXT-AMP-E-CHL** |
| **14** | **U311** | **x** | **x** | **I** | **4** | **32** | **4** | **128** | **1** | **0.25** | **1.25** | **S-CF-TET-AMP-E-CHL** |

Abbreviations: A, additivity; I, indifference; S, synergy; An, Antagonism; MIC, minimum inhibitory concentration; FIC, fractional inhibitory concentration; FICI, fractional inhibitory concentration index; CAR, carvacrol; S, streptomycin; CF, ciprofloxacin; TET, tetracycline; SXT, trimethoprim/sulfamethoxazole; AMP, ampicillin; E, erythromycin; CHL, chloramphenicol. Interpretation criteria (EUCAST 2000): synergistic (FICI ≤ 0.5), additive (0.5 < FICI ≤ 1), indifferent (1 < FICI ≤ 2), antagonistic (FICI > 2). Strains selected for the gene expression experiments are highlighted in bold.
